# Disciplinary barriers need communication: a behavioral and fNIRS study under group decision-making paradigm shift based on cabin design

**DOI:** 10.3389/fnins.2025.1594111

**Published:** 2025-05-20

**Authors:** Jiapeng Yang, Zuhua Jiang, Kexin Cheng, Lebao Wu

**Affiliations:** School of Mechanical Engineering, Shanghai Jiao Tong University, Shanghai, China

**Keywords:** interdisciplinary group decision-making, decision quality, functional near-infrared spectroscopy, decision paradigm shift, cognitive load

## Abstract

In the field of interdisciplinary engineering design, the terminology used by decision-makers from different disciplinary backgrounds often exhibits significant disciplinary heterogeneity, resulting in misunderstandings or communication barriers for decision-making teams. Due to the ambiguity of cognitive structures, the impact of interdisciplinary knowledge on decision-making quality and cognitive load was poorly answered. This study, grounded in utility theory and multi-criteria decision theory, introduced an enhanced multi-attribute decision-making task (MADM-LGD) to research the behavioral characteristics of decision-making groups and the cognitive shifts that occur during interdisciplinary decision-making paradigm transitions. An experiment utilizing Functional Near-Infrared Spectroscopy (fNIRS) was conducted based on a ship cabin design task, aiming to explore the neural mechanisms underlying interdisciplinary group decision-making. The analysis of experiment revealed several key findings: (1) Prior cognitive level does not significantly affect decision quality during the individual decision-making phase, but it positively influences decision quality during the group decision-making phase. (2) Interdisciplinary communication ability positively impacts decision quality. Hence, teams which exhibit stronger interdisciplinary communication achieve superior decision performance; (3) The task-oriented phase imposes a higher cognitive load compared to the non-task-oriented phase, while interdisciplinary communication helps alleviate this cognitive load, reducing the cognitive pressures associated with heterogeneous engineering semantics, promoting mutual understanding across disciplines, and ultimately enhancing decision quality. This study offers valuable guidance for advancing the empirical theories and practices of interdisciplinary group decision-making in artificial intelligence (AI) and human intelligence (HI).

## 1 Introduction

In the context of rapid advancements in technological innovation, decision-making tasks in the field of engineering product design have progressively evolved into interdisciplinary group decision-making tasks involving multiple objectives and knowledge domains (Wang et al., 2024). For group decision-making tasks in interdisciplinary fields, significant differences in knowledge structures across disciplines often lead to ambiguity and misunderstandings of specialized terms and concepts among decision-makers from different disciplines (Ju et al., 2016), which may result in communication barriers and adversely affect the quality of final decisions (Cooley, 1994). Therefore, studying how disciplinary heterogeneity impacts decision quality is of great significance for exploring the mechanisms of interdisciplinary group decision-making, reducing team communication barriers, and improving the efficiency of group decision-making.

However, existing research predominantly focuses on subjective emotional tendencies of decision-makers (Clark and Patrick, 2022) and qualitative studies at the managerial level (Masaryk, 2014; Intezari et al., 2016), with limited exploration into the quantitative analysis of the mechanisms underlying interdisciplinary group decision-making processes and the factors influencing decision quality, particularly in terms of decision-makers’ neurocognitive load and objective decision-making behaviors.

Due to the ambiguity in the mechanisms of group decision-making process, assessing the quality of group decisions solely through subjective cognitive factors is one-sided. The impact of group cognitive structures and the interdisciplinary heterogeneity of engineering semantics on the decision-making process, and ultimately on the quality of group decisions, has not been well answered. Moreover, there is limited direct experimental evidence on the cognitive structures associated with interdisciplinary group decision-making processes. To address this gap, this study proposes two cognitive indicators: “prior cognitive level” and “interdisciplinary communication capability.” Using ship cabin design as one classic case, this study explores the mechanisms of interdisciplinary engineering design practices and how two cognitive indicators designed influence the quality of group decision-making.

With advancements in cognitive science and neuroimaging technologies, the investigation of neural mechanisms underlying decision process has become increasingly feasible (Herd et al., 2021). Functional Near-Infrared Spectroscopy (fNIRS) has emerged as a promising non-invasive neuroimaging technique for capturing cortical hemodynamic activity (Fishburn et al., 2014). Within the field of cognitive psychology, fNIRS has proven effective for examining the neurophysiological foundations of complex cognitive processes (Herold et al., 2018; Kumar et al., 2017; Dai et al., 2024). Furthermore, fNIRS has demonstrated its sensitivity in detecting group-level differences in cognitive load and cortical activation (Ferrari and Quaresima, 2012; Pinti et al., 2018). Therefore, this study will utilize fNIRS for experimental data collection.

This study focuses on the neuroscientific basis of group decision-making process and the analysis of subject behavior under the interdisciplinary group decision-making process. Reviewing the relevant literature, Section 2 discusses the characteristics of interdisciplinary group decision-making, the factors affecting interdisciplinary group decision-making, and the design of the fNIRS experimental paradigm. Section 3 designs an improved multi-attribute decision transfer paradigm (MADM-LGD) and took the design of a luxury cruise ship cabin as an example and design an experiment of a new decision paradigm transfer based on fNIRS. Section 4 presents the statistical analysis results of the experiment, revealing how prior cognitive level and interdisciplinary communication capability during the decision-making process influence decision quality. The differences in group cognitive load under various decision-making conditions are also illustrated. Section 5 discusses the implications and contributions of the proposed decision transfer paradigm, making some recommendations for collaborative multi-agent decision-making and knowledge-intensive firms. Conclusions and future work are given in Section 6.

## 2 Related works

### 2.1 Characteristics of interdisciplinary group decision-making in the field of engineering

Interdisciplinary teams bring together expertise from various domains, facilitating comprehensive analysis and innovative solutions to complex engineering problems (Wisnioski et al., 2019). The diversity within these teams necessitates effective communication strategies to ensure clear understanding and information sharing among members with different backgrounds. Engineering decisions often involve balancing technical, economic, and social considerations, requiring a collaborative approach to navigate the multifaceted nature of these challenges. The convergence of multiple disciplines fosters an environment where creative ideas can emerge, leading to innovative engineering solutions (Olawale et al., 2018). Lai et al. (2019, 2020) found, through accident analysis and multi-intelligence modeling, that there is a mental model inconsistency between pilots and air traffic controllers in the process of aviation “unstable approach,” which is manifested in the understanding of the mission objectives, procedural execution, and risk perception bias. This mental modeling disconnection can easily lead to communication barriers and operational conflicts in high-pressure or dynamic environments, thus increasing flight risks. Multi-intelligence simulations further revealed that these cognitive biases may be amplified over multiple rounds of interaction, creating systemic hazards. The study emphasizes the importance of training and system design to improve the consistency of mental models of both parties to enhance synergy and flight safety. This suggests that mental model inconsistency is an important potential factor contributing to unstable approaches in aviation, and that training and system design are needed to enhance consensus and communication between pilots and controllers. In summary, interdisciplinary group decision-making in engineering involves the integration of diverse expertise, effective communication, complex decision processes, innovation, and a commitment to ethical and social considerations.

### 2.2 Factors affecting decision-making in interdisciplinary groups

Interdisciplinary groups play a critical role in addressing complex engineering, scientific, and societal challenges by integrating diverse expertise and perspectives. However, decision-making in such groups is influenced by several key factors, including team diversity, communication strategies, cognitive and social dynamics, and the interplay between technical and non-technical considerations.

The heterogeneity of interdisciplinary teams fosters innovation and creativity but also introduces challenges in aligning perspectives and priorities. Research highlights that diversity in cognitive styles, knowledge domains, and cultural backgrounds can significantly enhance decision quality when managed effectively (Wisnioski et al., 2019). Effective communication is vital to bridge gaps between disciplines. Frameworks like SUIT (Share, Understand, Integrate, and Team Decision) have been shown to reduce conflict and improve decision-making outcomes in engineering teams (Hoffart et al., 2015). Cognitive factors, such as shared mental models and team reflexivity, influence decision-making by fostering alignment and understanding among group members. Studies emphasize that reflexivity promotes strategic planning and long-term decision effectiveness (Weger et al., 2022). The convergence of technical and social considerations in engineering decisions necessitates frameworks that integrate diverse inputs while maintaining a focus on ethical and societal implications. Programs like DesignSpine (Olawale et al., 2018) have demonstrated success in preparing students for interdisciplinary decision-making environments. Leadership styles and conflict resolution strategies significantly influence group dynamics. Constructive controversy and the management of task-related debates while minimizing interpersonal tensions have been linked to higher team performance (Korb et al., 2015).

### 2.3 Experimental paradigms of group decision-making

Group decision-making in interdisciplinary engineering projects leverages various paradigms to address complex, multi-dimensional challenges. Dynamic decision-making focuses on sequential decisions influenced by real-time feedback and evolving system states. While effective in modeling real-world uncertainties and iterative adjustments, it lacks the capability to systematically evaluate competing objectives or integrate diverse disciplinary inputs critical for engineering design (Brehmer, 1992). Similarly, experimental games excel in analyzing cooperation and strategic behaviors within groups by simulating interactive decision-making scenarios. However, they often emphasize competitive dynamics and fail to offer structured approaches for multi-criteria optimization, making them less suited for collaborative engineering contexts where balancing diverse goals is crucial (Van Dijk and De Dreu, 2020). The Analytical Hierarchy Process (AHP) is a structured decision-making method that decomposes complex problems into hierarchical levels, allowing decision-makers to evaluate alternatives based on pairwise comparisons of criteria (Saaty, 2018). Its main strength lies in handling subjective judgments, which are converted into numerical priorities. However, AHP is less effective in scenarios with many criteria or alternatives due to consistency issues in pairwise comparisons.

By contrast, Multi-Attribute Decision-Making (MADM) stands out as a robust paradigm that addresses these limitations effectively. MADM methods such as the Weighted Sum Method (WSM) and PROMETHEE are specifically designed to evaluate alternatives across multiple attributes, enabling a comprehensive trade-off analysis between objectives like performance, cost, and sustainability (Sun et al., 2016). Unlike other paradigms, MADM seamlessly integrates expert opinions, fostering consensus in interdisciplinary teams by combining diverse perspectives and resolving conflicts. Furthermore, its ability to incorporate fuzzy set theory and grey system theory equips it to handle uncertainty and incomplete information, a common challenge in engineering projects (Sun et al., 2016). The transparency and traceability of MADM processes, such as weight calculation and scoring, enhance its validity and facilitate stakeholder acceptance, making it a preferred choice for multi-objective interdisciplinary tasks.

### 2.4 Applications for fNIRS in the field of cognitive processing

Higher cognitive ability is a more complex cognitive ability of an individual, which is mainly responsible for memory, language, planning, decision-making, and executive functioning (Boschin et al., 2017), and the development of these higher cognitive abilities is closely related to the prefrontal cortex (PFC) (Mack et al., 2020). fNIRS is an effective technique to monitor the activity of the prefrontal cortex. fNIRS has been used in different fields to detect the changes of blood oxygen level in the prefrontal region by fNIRS technique and has obtained pioneering discoveries (Herff et al., 2014; Herff et al., 2013; Wang et al., 2021). fNIRS has emerged as a non-invasive neuroimaging tool in the last decade and has been widely used to monitor brain blood oxygenation activity during cognitive tasks. fNIRS, as a flexible and effective brain imaging tool, has shown a wide range of applications in cognitive process research. For cognitive load assessment, researchers combined fNIRS and eye tracking techniques to analyse blood oxygenation changes and eye movement behaviors in the prefrontal cortex under different task difficulties. The results showed that the activity of prefrontal cortex increased significantly with increasing task difficulty, and the pupil diameter enlarged accordingly, indicating that fNIRS can effectively monitor changes in cognitive load (Yu et al., 2024). In addition, fNIRS has played an important role in social cognition research. By measuring the brain activity of multiple individuals simultaneously, researchers have explored the phenomenon of neural synchronization in interpersonal interactions and gained a deeper understanding of the cognitive mechanisms involved in social interactions (Wang et al., 2023).

In contrast to other tools for monitoring neural activity shown in Table 1 despite the convenience of EEG recordings, its poor spatial resolution limits its ability to identify neural substrates involved in complex behaviors involving the co-activation of multiple cortical regions (Scholkmann et al., 2014). fMRI is very costly to run and is often uncomfortable for study participants and therefore cannot be adapted to the prolonged cognitive processes involved in engineering problem solving and design. In addition, due to its low temporal resolution, fMRI does not allow for longer sustained assessment of blood oxygen levels in brain regions during engineering design activities. In contrast, fNIRS has emerged as a new neuroimaging analysis tool that allows participants to comfortably use it while operating equipment as well as performing engineering design tasks. Typically, engineers perform most high-level cognitive behaviors (e.g., group discussion and decision-making) with some subconscious movements of the limbs or head, and head-movement-sensitive techniques such as EEG and fMRI, which require high head-movement, are difficult to implement, whereas the fNIRS technique is more tolerant to the head movements of subjects in the experiment. Secondly, the fNIRS technique can be used for cognitive load detection (Fishburn et al., 2014), and it can completely cover the prefrontal lobe and the surrounding brain regions in terms of spatial localization, and has high temporal and spatial resolution, which is a great advantage in detecting the neurocognitive mechanisms of prefrontal-related cognitive abilities (Szczepanski and Knight, 2014), and can meet the needs for time course and spatial localization in the interdisciplinary group decision-making experimental task of this paper.

**Table 1:** Comparison of neurocognitive tools.

Indicator	EEG	fMRI	fNIRS
Temporal resolution	High	Low	High
Size	Medium	Large	Small
Cost	Low	Expensive	Moderate
Cognitive load	None	Present	Present
Portability	Low	None	High
Subject position	Sitting	Lying down	Dynamic or static
Motion interference	Strong	Strong	Weak
Electromagnetic resistance	Medium	Low	High
Spatial resolution	Low	High	Medium
Head sensitivity	Medium	High	Low

### 2.5 Decision-theoretic pathways

In cross-system or interdisciplinary contexts, semantic heterogeneity often leads to inconsistencies in concept definitions and divergent interpretations of information, which becomes a significant source of cognitive conflict. Scholkmann et al. (2014) pointed out that semantic coordination requires negotiation of concept meanings across semantic models, where heterogeneity essentially manifests as semantic conflict. In decision support environments, inconsistencies in information interpretation and priority ranking by individuals or groups directly reflect cognitive conflict (Sengupta, 1993). To resolve and reconcile such conflicts, individuals must invest additional cognitive resources in information alignment and negotiation, which undoubtedly increases cognitive load. Previous studies have observed that the handling of conflicting information significantly raises the burden on working memory and complicates decision-making in tasks such as team collaboration and diagnostic processes (Fiore, 2013). Furthermore, Achtziger et al. (2020) found that under high cognitive load conditions, although reaction times decrease, decision-making biases become more pronounced due to interference with rational judgment abilities. To alleviate these negative effects, the establishment of shared understanding is considered to play a critical regulatory role. Bouquet et al. (2004) showed that by constructing formal semantic mappings, cognitive conflict and coordination costs can be effectively reduced, promoting consistency in information interpretation. Based on the above literature, we propose the following theoretical path model: semantic heterogeneity leads to differences in semantic understanding, which in turn triggers cognitive conflict, increases working memory usage and cognitive load, ultimately resulting in intuitive decision-making biases and decreased communication efficiency. This model not only incorporates cognitive load theory, which suggests that limited working memory is prone to overload when facing complex or conflicting information but also integrates shared understanding theory and semantic coordination mechanisms, providing a solid theoretical foundation for further exploration of the role of semantic heterogeneity in the decision-making process.

### 2.6 Summary

In the field of engineering design, decision-making tasks typically require collaboration among multidisciplinary teams. Differences in knowledge structures across various disciplines can lead to ambiguities or even misunderstandings of specialized terminology and concepts, thereby creating communication barriers that ultimately affect the quality of final decisions. Although the management and psychology literature has discussed several factors influencing group decision-making, most studies have focused on management theories and behavioral analyses, paying insufficient attention to the cognitive mechanisms and neural underpinnings of complex interdisciplinary group decision-making processes.

To address this gap, this paper constructs the decision path model “Semantic Heterogeneity → Differences in Semantic Understanding → Cognitive Conflict → Increased Working Memory Usage & Cognitive Load → Intuitive Decision-Making Biases & Decreased Communication Efficiency.” and uses a multi-attribute decision-making task (MADM-LGD) in cruise ship cabin design as a case study to investigate how two key cognitive indicators—prior cognitive level and interdisciplinary communication capability—affect the quality of group decision-making. The study employs functional near-infrared spectroscopy (fNIRS) to monitor blood oxygen dynamics in the prefrontal cortex. By collecting participants’ brain oxygenated hemoglobin data along with behavioral semantic data and applying natural language processing and statistical analysis techniques, the research reveals changes in group cognitive load under different decision-making paradigms and examines their relationship with decision outcomes.

By proposing four hypotheses, this study aims to elucidate the impact of cognitive structures and communication mechanisms on decision quality in interdisciplinary group decision-making, thereby providing theoretical foundations and empirical support for collaborative multi-agent decision-making in the field of engineering design.

*H1*: Different decision technological paradigms lead to significantly different quality of group decision-making.

*H2*: Prior cognitive level is significantly correlated to the quality of decision-making.

*H2a*: Prior cognitive level is significantly correlated to the quality of individual decision-making.

*H2b*: Prior cognitive level is significantly correlated to the quality of group decision-making.

*H3*: Interdisciplinary communicative capability is significantly correlated to the quality of group decision-making.

*H4*: The cognitive load of different decision technological paradigms is significantly different.

*H4a*: There was a significant difference in cognitive load between the task guidance and non- task guidance phases within the individual and group decision-making phases.

*H4b*: There was a significant difference in cognitive load in the task guidance phase between the individual and group decision-making phases.

H1 mainly aims to explore differences in group decision-making performance under different decision-making paradigms. After clarifying the differences in performance under these paradigms, H2 further investigates whether groups with varying levels of subjective prior knowledge exhibit significantly different group decision-making performances across different decision-making paradigms. H3 delves deeper from an objective behavioral perspective, examining whether differences in objective interdisciplinary communication reflect the subjective prior knowledge of participants, and explores if groups with varying levels of interdisciplinary communication show significantly different decision-making performances under group decision-making paradigms. Finally, H4 investigates, from the perspective of neurocognitive load, the cognitive load variations occurring with changes in decision-making pathways, combining neurocognitive findings with behavioral indicators to corroborate the conclusions of H2 and H3.

## 3 Experimental methods

The data were extracted and utilized for analysis between July and August 2024. The research data were processed with privacy-protecting ID measures, ensuring that the information cannot be reverse-engineered to identify individual participants through the extracted data. To study how the factors of decision-making influence the decision quality, this study designed a modified multiple attribute decision making (MADM) process called MADM-LGD based on fNIRS. MADM-LGD consists of two experimental stages, the single discipline background individual decision-making stage and the interdisciplinary background group decision-making stage.

### 3.1 The overall procedure of the experiment

**Figure 1:**
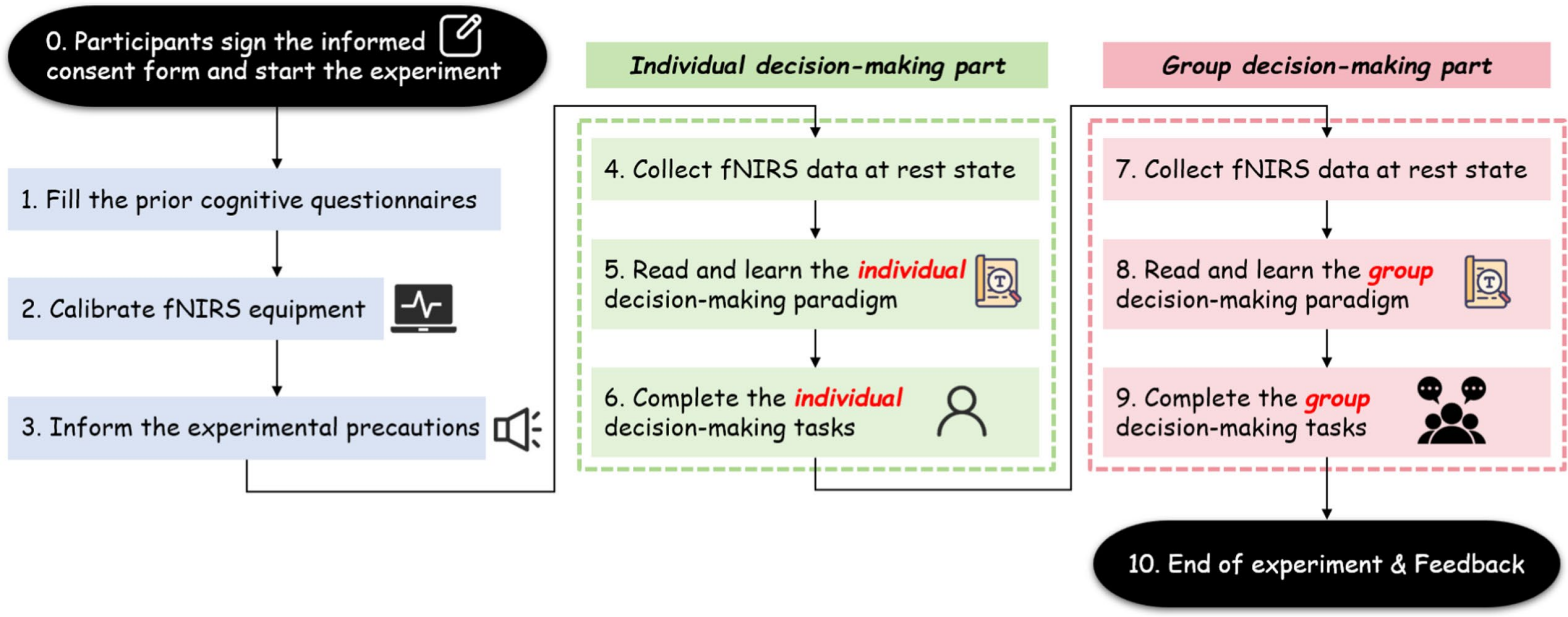
The procedure of the whole experiment.

The experiment of interdisciplinary group decision-making was performed in an independent and quiet laboratory environment. It consisted of two decision stages: an individual decision-making stage and a group decision-making stage. As shown in Figure 1, participants signed informed consents approved by the Institutional Review Board (IRB) of Shanghai Jiao Tong University in the preparation stage of the experiment. Anonymous necessary information was also collected in this stage, such as gender, age, left/right hand. Afterward, participants finished the prior cognitive questionnaires, and their basic cognitive levels were collected. In the individual decision-making stage, participants comprehended the decision materials and chose one best scheme individually. In the group decision-making part, participants discussed with each other and chose one best scheme jointly. Note that before the beginning of each experimental part, the participants should rest adequately to ensure that the brain Oxy-Hb concentration returned to the resting state level. After each phase of instrument calibration, the Oxy-Hb concentration data of the task state would be collected continuously.

**Figure 2:**
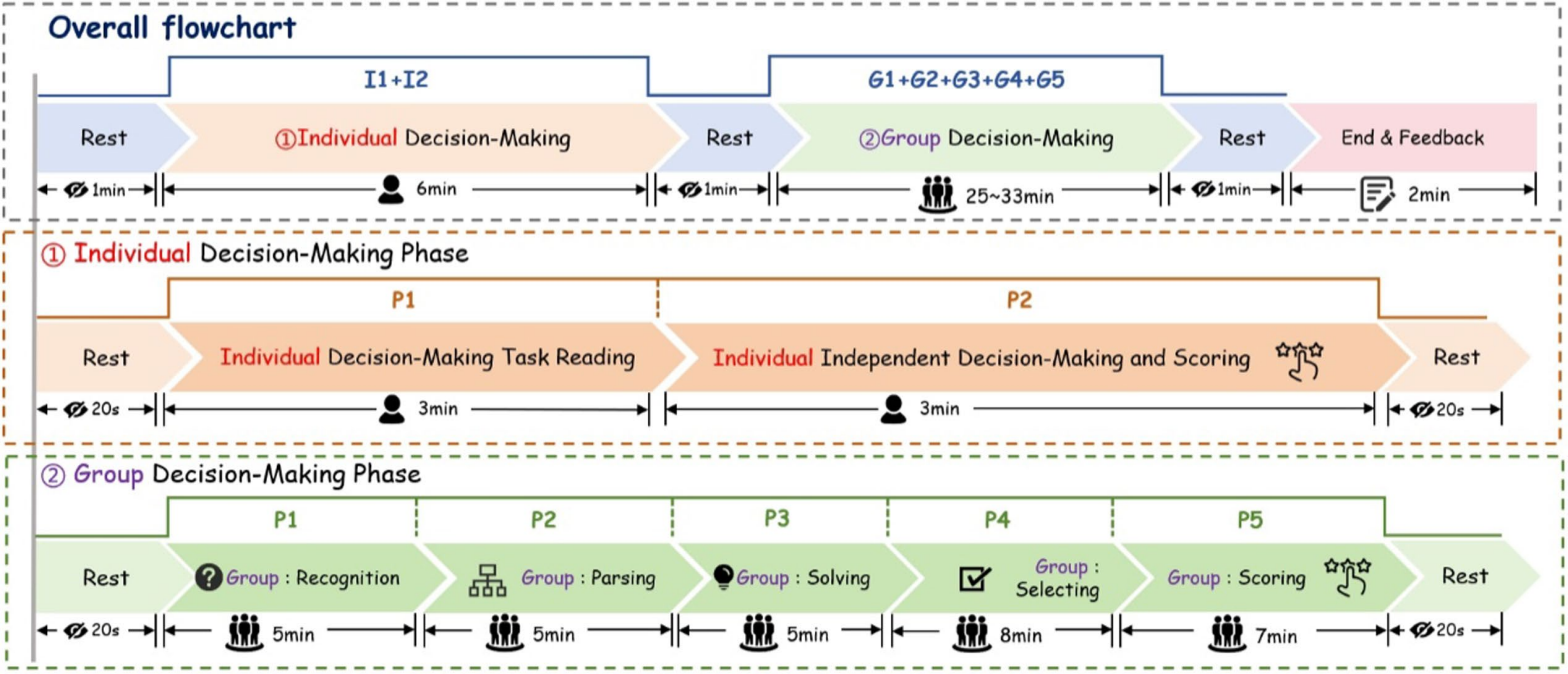
Stimulus sequences.

The whole experiment spent total about 45 min, and the stimulus sequences were shown in Figure 2.

During the individual decision-making phase, participants were first briefed on the research objectives, experimental tasks, and necessary precautions. Following a 20-s break, they reviewed the decision materials—which included design objects, design parameters, and design constraints. Subsequently, they independently scored and made their individual decisions. Both the reading and decision-making parts lasted for 3 min.

During the group decision-making phase, participants followed the process outlined in Figure 2 to conduct a leaderless group discussion (LGD) and jointly select a common scheme. In the recognition stage, they collaboratively analyzed the overall design task and decomposed it into several manageable sub-issues. During the parsing stage, they further deliberated on the detailed parameters of each scheme and clarified any ambiguous concepts. In the solving stage, the group compared the various schemes and addressed all the identified sub-issues. During the selecting stage, participants engaged in discussions to jointly choose the scheme deemed most effective. Finally, in the scoring stage, each participant provided an individual scoring matrix for each scheme after the group discussion.

### 3.2 Design of experimental paradigms

The cruise ship cabin design task is a typical multi-attribute decision-making task. The experiment used a multiple attribute decision-making task (MADM) (Sun et al., 2016) to consider a finite solution ranking or selection problem with multiple attributes. For interdisciplinary decision-making groups, this study introduced a group discussion phase based on the MADM task and proposes an interdisciplinary leadless group discussion (LGD) (Bouquet et al., 2003). MADM task paradigm in this study called MADM-LGD. Subjects are required to discuss the attributes of the options and the basis for selection with subjects from other disciplines using relevant knowledge from their own disciplines. According to the Concept-Knowledge theory (C-K theory) (Hjørland, 2009), the interdisciplinary technical focus of the decision-making task can be used to construct a cognitive structure in the minds of the subjects, and the think aloud method is adopted. The think aloud protocol (TAP) is effective in obtaining fragments of ideas in the subjects’ minds, and thus subjects are required to use the technical focus of their own disciplines (disciplinary terminology) to express themselves verbally during the group discussion phase (Fonteyn et al., 1993). To control the discussion content and discussion length in the group discussion, all subjects will be discussed according to the prompts provided by the master test, answer the decision-related questions such as design goals and design constraints, and be recorded and evaluated by the master test. The decision-making group will proceed with the individual decision-making paradigm followed by the group decision-making paradigm, the details of the two technical paradigms are shown in Table 2.

**Table 2:** Experimental paradigms.

Technological paradigm (TP)	Individual decision-making TP	Group decision-making TP
Participant	Single-discipline subject	A decision-making group consisting of three interdisciplinary subjects
Decision object	Luxury Cruise Ship Cabin Scheme Decision-making	
Goal	Reading materials, choosing one scheme that is recognized as the best one and scoring for each scheme.	
Constraints	Making decision independently	Following the MADM to make group discussion and decide jointly

### 3.3 Experimental data acquisition and processing

fNIRS data were collected under two conditions: (1) a resting state and (2) a decision-making task. During the resting state, participants sat quietly in a dimly lit room with their eyes closed and remained awake for 2 min, allowing for baseline physiological measurements.

**Figure 3:**
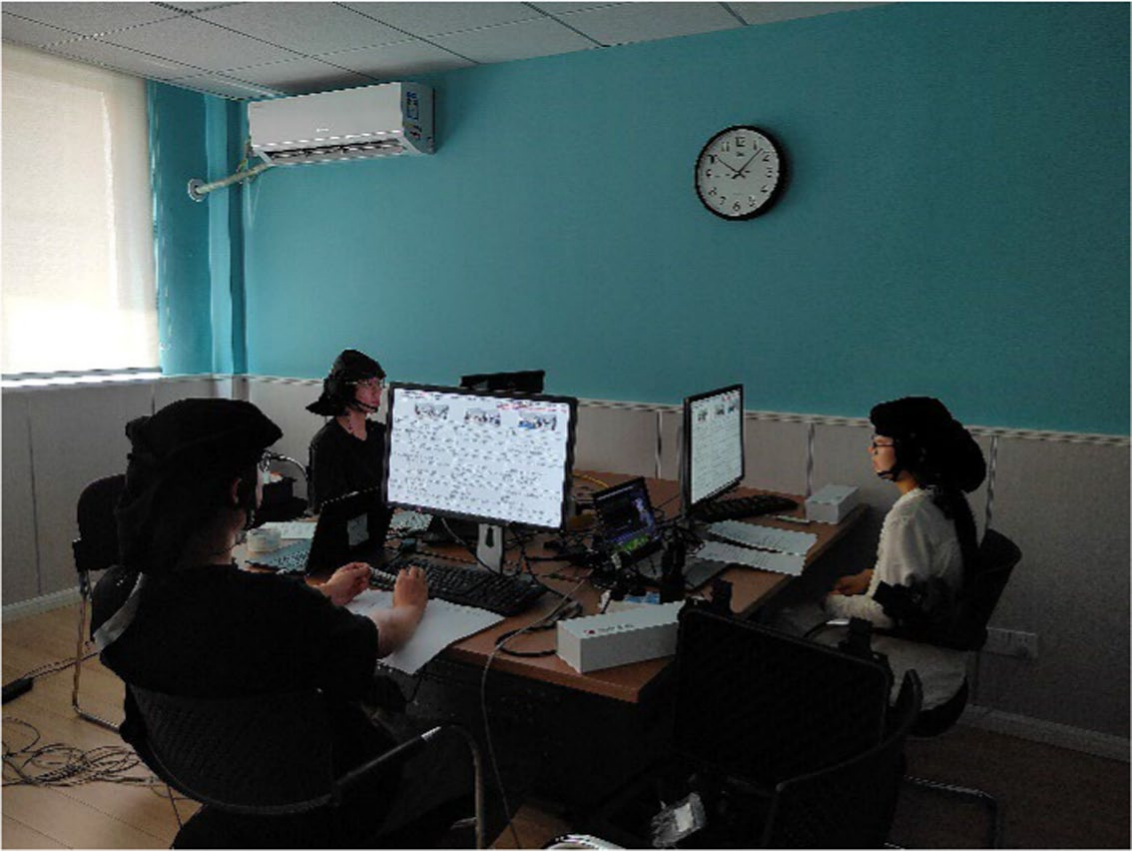
Layout of the experimental environment.

The experimental environment is laid out as shown in Figure 3. The experiment utilized the Cortivision Photon Cap (model C20), a portable near-infrared optical brain imaging system, along with the Cortivision Pathfinder to measure cortical hemodynamic activity. The Photon Cap adopted an enhanced version of the “10–5 system” (Oostenveld and Praamstra, 2001) for electrode placement, offering higher precision compared to the traditional “10–20” and “10–10” systems commonly used in EEG.

**Figure 4:**
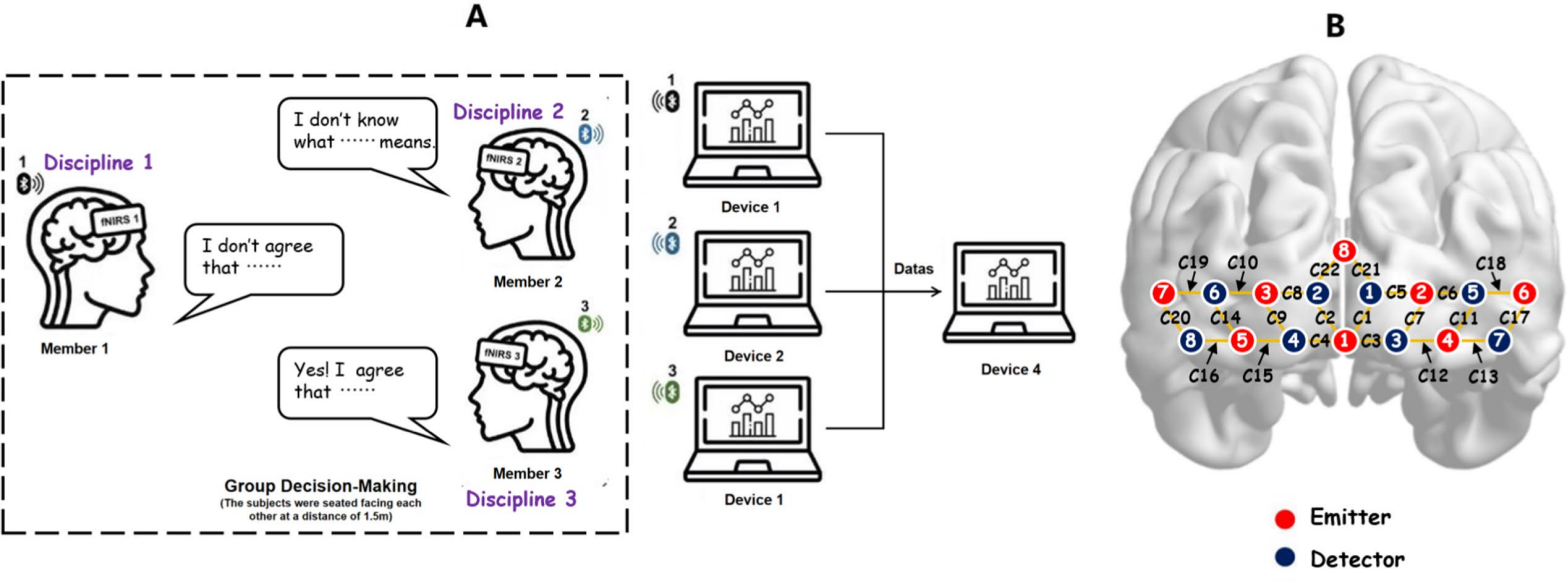
Experimental environment and layout of channels. **(A)** Schematic diagram of interdisciplinary group decision-making. **(B)** Layout of brain region probes and channels.

The setup included 22 channels targeting the prefrontal cortex, composed of 8 light sources and 8 detectors, with a sampling rate of 6–7 Hz. Figure 4 depicts the electrode arrangement: light sources are marked in red, detectors in blue, and fNIRS channels as yellow lines. Additional details regarding Brodmann areas, anatomical locations, regions of interest (ROIs), and associated channels are summarized in Table 3.

**Table 3:** regions of interest and associated channels.

Brain regions Channels (*Brodmann Areas*)		
10 FOA (frontopolar area)	Left:	C1, C3, C5, C6, C7, C12, C21
	Right:	C2, C4, C8, C9, C10, C15, C22
45 PTBA (pars triangularis Broca’s area)	Left:	C17
	Right:	C20
46 DLPFC (dorsolateral prefrontal cortex)	Left:	C11, C18
	Right:	C14, C19
47 IPFG (inferior prefrontal gyrus)	Left:	C13
	Right:	C16

The raw data collected by the fNIRS device was pre-processed through the NIRS_KIT (version 3.0_Beta) (Hou et al., 2021) toolbox of Matlab_R2022a software, which included: registering MNI coordinates, constructing GLM design matrix, low-pass filter based on hemodynamic response function (hrf), wavelet MDL detrending method, and temporal correlation estimation of Beta value. Then SPSS 25.0 software was used for statistical analysis of behavioral data and cortical blood oxygen data.

The following experimental raw data of participants was collected:

1. Gender, age, left/right hand (through survey questionnaire).
2. Engineering semantics-based dialogue texts generated by interdisciplinary group discussion.
3. The cortical Oxy-Hb concentration during MADM-LGD task.

### 3.4 Stimulus materials for group decision-making tasks

Luxury cruise ship cabin design exemplifies a typical Multi-Attribute Decision Making (MADM) task. In the interdisciplinary design process of cruise ship staterooms, participants from various disciplinary backgrounds tend to prioritize different attributes of the design. For instance, research indicates that individuals with a structural engineering background often emphasize functionality and practicality, focusing on elements such as efficient storage arrangements, optimal lighting, and window layout. In contrast, those with an environmental engineering background are more inclined to consider passenger comfort by prioritizing factors such as temperature control, air quality management, and noise isolation. Meanwhile, participants from an aesthetics background tend to focus on visual appeal and brand image. Additionally, for various types of cruise ships, whether family-oriented, luxury, or themed, the cabin design should align with and enhance the overall aesthetic by selecting appropriate colors, materials, and decorative elements.

In terms of the simulation of interdisciplinary knowledge integration, the experiment prompted subjects with different disciplinary backgrounds to have to integrate their expertise and work together to solve the design problem by simulating a real cruise ship cabin design task. This design simulates interdisciplinary collaboration in engineering practice and provides a near-realistic research environment for the study. The knowledge used in the experimental design process comes from the textbooks *Ship Aesthetics and Cabin Design* (Yang and Jing, 2021) and *Ship Cabin Environmental Engineering Research and Design* (Dongmei, 2020), and the decision-making materials are selected based on the following typical considerations: first, the completeness of the knowledge system. The book comprehensively introduces five major aspects of ship cabin design including internal cabin structure and layout, color environment, light environment, cabin insulation design (air environment), and noise environment, which ensures the comprehensiveness and systematicity of the experimental materials at the knowledge level; second, the integration of interdisciplinary knowledge. The book integrates the theories and practices of many disciplines such as aesthetics, ergonomics, environmental science, etc., which is highly compatible with the theme of interdisciplinary group decision-making in this study; third, the richness of practical cases. The application of ship aesthetics in actual ship modelling and cabin design is demonstrated through specific case studies, providing rich contextual simulation and discussion materials for experiments; fourth, the advanced nature of the design methodology. The methods of the textbook reflect the current frontier in the field of ship design, which helps to stimulate in-depth discussion among the subjects.

**Figure 5:**
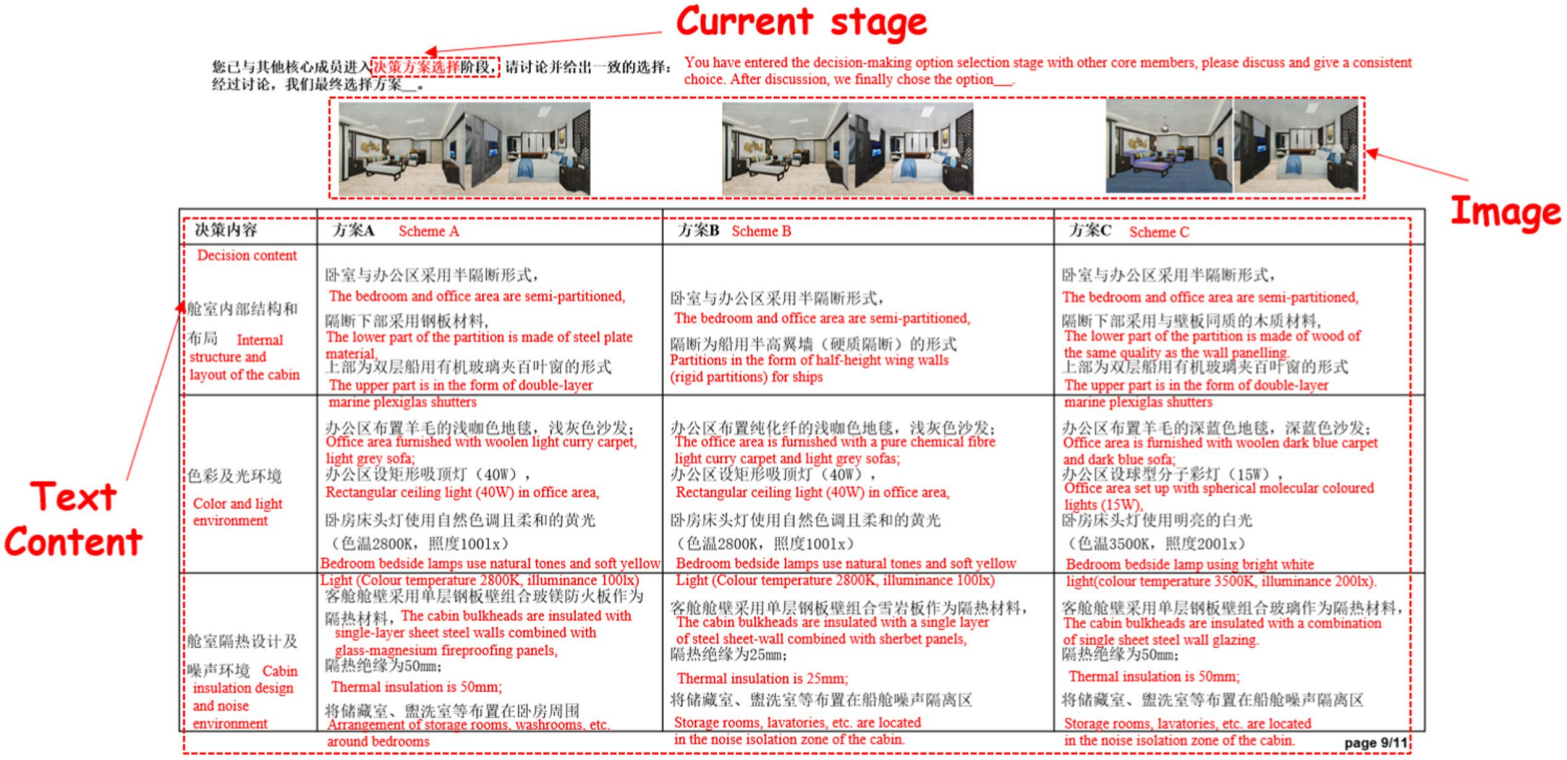
Stimulus materials.

According to the definition of the MADM task for cruise ship stateroom design, the experimental cases must meet the following requirements: (a) the case must be a multiple alternative selection or comparison problem; (b) each alternative must contain multiple different attributes for decision makers to make comparisons; and (c) the number of attributes must be consistent with the number of decision makers for the sake of ensuring the reasonableness and accuracy of the group scoring. Based on the above requirements, three design options (as shown in Figure 5) are selected from the ‘Design Example of a VIP Suite on a Roll-on Roll-off Ship’ provided by the *Ship Cabin Environmental Engineering Research and Design* (Dongmei, 2020) for evaluation and selection by the decision-making team of the interdisciplinary group. In the guest cabin design case, five design aspects are considered: space planning and layout, color environment, light environment, air environment, and noise environment. However, since the MADM task for cruise ship stateroom design draws on expertise from only three disciplines—structural engineering, aesthetics, and environmental sciences—the attributes of the interdisciplinary group decision-making alternatives have been consolidated into three clearly defined and operationalized categories for this experiment: (1) *Cabin internal structure and layout*, encompassing design factors such as spatial arrangement, structural integrity, and partition materials that directly relate to structural engineering concerns; (2) *Color and light environment*, focusing on visual aesthetics, color coordination, lighting intensity, and illumination comfort that influence occupants’ visual perceptions and overall aesthetic experience; and (3) *Cabin insulation design and noise environment*, addressing environmental science aspects, including thermal insulation effectiveness, acoustic materials selection, and the control of noise and vibration levels to ensure occupant comfort and environmental quality.

In terms of decision consensus and decision conflict design, in the cruise ship stateroom design MADM task, which requires interdisciplinary teams with backgrounds in structural engineering, aesthetics, and environmental science to design a ship’s stateroom, the decision-making task is for three people to work together to examine the feasibility of the three design alternatives shown in Figure 5 (denoted as Option A, Option B, and Option C, respectively), and to give the team’s final choice of one option. Since the number of alternatives is greater than the number of final choices, it meets the requirement for group decision-making consensus. To ensure that decision-makers from different disciplines both engage in cognitive conflict and reach consensus, all three design options have been crafted to incorporate interdisciplinary elements across the three attribute dimensions. For instance, in the attribute dimension of internal structure and layout of the compartment, one criterion— “the material of the partitions must be identical to that of the wall panels”—is grounded in structural engineering principles, which tends to lead structural engineering decision-makers to favor Option B or Option C. In contrast, the criterion “half-height wing wall,” a distinctive technical feature of structural engineering, may present cognitive challenges for decision-makers in aesthetics and environmental engineering, thereby fostering cognitive conflict. Within this dimension, Option C is the closest to the standard alternative.

### 3.5 Definition for experimental variables

Table 4 shows the notation of symbols of this study. To quantify these aspects of research factors (prior cognitive level of decision-making, Decision quality, and Capability of interdisciplinary communicative competence), we separately processed the experimental data as follows.

**Table 4:** Notation of symbols of this study.

Notation	Explanation
$P_{\text{co}}$	Prior cognitive level of decision-making
$Q_{d}$	Decision quality
O	The scoring matrix derived from the group’s joint decision-making
R	The standard scoring matrix of the standard case
K	The number of disciplines or the number of participants in one decision group
N	The number of stages in group decision-making of MADM
$P^{\left(k\right)}$	The individual scoring matrix of the k-th participant
$C_{\text{inter}}$	Interdisciplinary communicative capability
H	The high-centricity semantic set
$D_{k}$	Semantic set for discipline k
$P_{n}$	A semantic repository set for n-th stage in group decision-making
L	The language sequence of the whole group in group discussion
$L_{n}^{\left(k\right)}$	The language sequence of k-th participant in n-th stage group discussion
$S_{k}$	The capacity of sharing his/her knowledge in communication for k-th participant
$A_{k}$	The capacity of absorbing other disciplines’ knowledge in communication for k-th participant
$I_{j}$	The stage j of individual decision-making phase; $I_{1}$: the reading and comprehension stage of individual decision-making; $I_{2}$: the scheme selection and scheme scoring stage of individual decision-making
$G_{j}$	The stage j of group decision making phase; $G_{1}$: the cognitive stage of group decision-making; $G_{2}$: the analysis stage of group decision-making; $G_{3}$: the solving stage of group decision-making; $G_{4}$: the scheme selection stage of group decision-making; $G_{5}$: the scheme scoring stage of group decision-making

Independent variables: (1) Prior cognitive levels for decision-making tasks in engineering design; (2) Interdisciplinary communicative capability in group decision-making tasks.

Dependent variables: (1) Quality of decision-making at different decision stages; (2) Cognitive load under different decision stages.

#### 3.5.1 Prior cognitive level of decision-making ($P_{\text{co}}$)

The prior cognitive level refers to the knowledge and methods individuals have already mastered before encountering a specific technological challenge (Ifenthaler et al., 2011). To quantify the prior cognitive level of decision-making in this paper, before the experiment, each participant will complete a survey related to decision-making cognition, and the quantified results of the survey $P_{\text{co}}$ will serve as an indicator to measure the validity and integrity of decision-making cognitive structures.

#### 3.5.2 Decision quality ($Q_{d}$)

Decision quality refers to the contribution of a decision to reaching organizational goals (Dowie and Kaltoft, 2020). In this study, decision quality is defined as the cosine similarity between the decision scoring matrix and the standard scoring scheme matrix, and higher similarity indicates better decision quality.

Specifically, there are two matrices, O and R. Matrix O represents the scheme scoring matrix derived from the group’s joint decision-making, while matrix R represents the standard scheme scoring matrix. And $O_{\text{ij}}\in O\;(1⩽i⩽n,1⩽j⩽m)$ and $R_{\text{ij}}\in R\;(1⩽i⩽n,1⩽j⩽m)$ are the elements of corresponding scoring matrices. Thus, using the cosine similarity, the decision quality $Q_{d}$ of a certain group is defined with Equations 1, 2.


(1) $$Q_{d}=\text{Sim}(O,S)=\frac{O\cdot R}{∥O∥∥R∥}=\frac{\sum_{i=1}^{n}\sum_{j=1}^{m}O_{\text{ij}}R_{\text{ij}}}{\sqrt{\sum_{i=1}^{n}\sum_{j=1}^{m}O_{\text{ij}}^{2}}\sqrt{\sum_{i=1}^{n}\sum_{j=1}^{m}R_{\text{ij}}^{2}}}$$

(2) $$O_{\text{ij}}=\frac{\sum_{k=1}^{K}P_{\text{ij}}^{\left(k\right)}}{K}$$

Where $P^{\left(k\right)}$ is the individual scoring matrix of the k−th participant and K is the number of participants in one decision group.

#### 3.5.3 Interdisciplinary communicative capability ($C_{\text{inter}}$)

For interdisciplinary group decision-making, a diverse disciplinary background does not necessarily lead to decision-making gains (Brignol et al., 2024). On the contrary, high levels of disciplinary heterogeneity may hinder conceptual understanding (Brignol et al., 2024). To address the barriers of interdisciplinary collaboration, teams must engage in thorough communication to clarify and explain the engineering semantics and concepts of different disciplines. This facilitates the integration of knowledge within the team and enhances the quality of group decisions.

This study posits that effective team decision-making processes require comprehensive communication. Accordingly, individual decision-makers representing distinct disciplines should not only articulate and elucidate the technical semantics of their own fields which are known as output sharing but also actively engage in comprehending the technical semantics of other disciplines which are called input comprehension. Based on this premise, we propose an interdisciplinary communication capability indicator, $C_{\text{inter}}$, designed to quantify a team’s ability to mitigate disciplinary heterogeneity and foster mutual understanding.

This study defines effective interdisciplinary communication as the process in interdisciplinary team decision-making where team members from different disciplines communicate using the specialized terminology from their respective fields. Each discipline possesses its own corpus consisting of specific domain vocabulary. In our $C_{\text{inter}}$ index, only interactions involving vocabulary from these disciplinary corpora during interdisciplinary team communication are considered effective interdisciplinary exchanges. Conversational fillers such as “emm” or non-discipline-focused phrases like “This is not good.” are excluded from the analysis of interdisciplinary communication content.

This study defines a high-centricity semantic repository set including engineering semantics that occurred frequently, notated by H (h∈H). There are K kinds of disciplines (K participants-one group) in the experiment and each discipline has its semantic repository set $D_{k}$ ($d_{k}\in D_{k})$. And there are N states in group decision-making phase and each decision-making phase of the MADM paradigm owns its semantic repository set $P_{n}$ ($p_{n}\in P_{n}$). There are several examples of semantic repository sets shown in Table 5.

**Table 5:** Several examples of semantic repository set.

Engineering semantic library	Several examples
High frequency semantic library	{‘cabin’, ‘Chinese style’, ‘comfortable’, …}
Ship structure semantic LIBRARY	{‘structural strength’, ‘stability’, …}
Aesthetic design semantic library	{‘color temperature’, ‘beautiful’ …}
Environmental engineering semantic library	{‘vibration’, ‘lighting’, …}
G1: cognitive stage semantic library	{‘features’, ‘subproblem’, …}
G2: analysis stage semantic library	{‘parameters’, ‘share’, …}
G3: solving stage semantic library	{‘comparison’, ‘difference’ …}
G4: scheme selection semantic library	{‘best’, ‘better’, ‘because’, …}
G5: scheme scoring semantic library	{‘scoring’, ‘full marks’, ‘minus’, ‘plus’ …}

In Equation 3, L means the language sequence of the whole group in group discussion. $L_{n}^{\left(k\right)}$($l_{n}^{\left(k\right)}\in L_{n}^{\left(k\right)}$) means the language sequence of k-th participant in n-th stage group discussion. Sentence $l_{n}^{\left(k\right)}$ consists of a set of sequential words $l_{n}^{\left(k\right)}=\{w_{1},w_{2},\ldots \ldots \}$.


(3) $$\begin{array}{c} L=\overset{N}{\underset{n=1}{\cup }}\overset{K}{\underset{k=1}{\cup }}L_{n}^{\left(k\right)} \end{array}$$

$S_{k}$ means the capacity of k-th participant sharing his/her discipline knowledge in communication, which is defined with Equation 4.


(4) $$\begin{array}{c} S_{k}=\frac{\sum_{n=1}^{N}\text{Match}(L_{n}^{\left(k\right)},H\cup D_{k}\cup P_{n})}{\sum_{n=1}^{N}\text{Match}(L_{n}^{\left(k\right)},H\cup (\overset{K}{\underset{k=1}{\cup }}D_{k})\cup P_{n})} \end{array}$$

$A_{k}$ means the capacity of k-th participant absorbing other disciplines’ knowledge in communication, which is defined with Equation 5.


(5) $$\begin{array}{c} A_{k}=\frac{\sum_{n=1}^{N}\text{Match}(L_{n}^{\left(k\right)},H\cup (\overset{K}{\underset{\begin{array}{c} i=1\\ i\neq k \end{array}}{\cup }}D_{i})\cup P_{n})}{\sum_{n=1}^{N}\text{Match}(L_{n}^{\left(k\right)},H\cup (\overset{K}{\underset{k=1}{\cup }}D_{k})\cup P_{n})} \end{array}$$

The operator Match(L,S) counts the number of occurrences of the elements of the set S in the language sequence L.

Drawing inspiration from the design of the F-score (Powers, 2011), $C_{\text{inter}}^{\left(k\right)}(\beta )$ is defined by Equations 6, 7.


(6) $$\begin{array}{c} \frac{1}{C_{\text{inter}}^{\left(k\right)}(\beta )}=\frac{1}{1+\beta^{2}}(\frac{1}{S_{k}}+\frac{\beta^{2}}{A_{k}}) \end{array}$$

(7) $$\begin{array}{c} C_{\text{inter}}^{\left(k\right)}(\beta )=(1+\beta^{2})\frac{S_{k}*A_{k}}{\beta^{2}S_{k}+A_{k}} \end{array}$$

For a single decision-making individual, $C_{\text{inter}}^{\left(k\right)}$ is an indicator defined as the comprehensive capacity both the capacity of sharing his/her knowledge in communication and the capacity of absorbing other disciplines’ knowledge in communication. When β>1, $A_{k}$ has a greater impact, and the indicator places more emphasis on the participants’ ability of absorbing other disciplines’ knowledge; When β<1, $S_{k}$ has a greater impact, and the indicator pays more attention to the participant’s ability to share her/his knowledge.

Let β=1, it means $S_{k}$ and $A_{k}$ are given equal importance (shown in Equation 8).


(8) $$\begin{array}{c} C_{\text{inter}}^{\left(k\right)}(\beta =1)=2*\frac{S_{i}*A_{i}}{S_{i}+A_{i}} \end{array}$$

For a whole cluster decision-making group, we take the average to get the indicator $C_{\text{inter}}$ with the Equation 9. K means the number of participants in one decision group.


(9) $$\begin{array}{c} C_{\text{inter}}=\frac{\sum_{k=1}^{K}C_{\text{inter}}^{\left(k\right)}(\beta =1)}{K} \end{array}$$

#### 3.5.4 Cognitive load under the group decision-making

Cognitive load is generally influenced by three factors: the complexity of knowledge within the learning materials, the organizational rules or presentation methods of these materials, and the learner’s prior experience. In this study, all participants received identical learning materials, ensuring consistency in both complexity and organization.

fNIRS technology has been extensively applied in detecting cognitive load. Among several fNIRS indicators, changes in Oxy-Hb concentration are particularly significant (Maidan et al., 2015). HbO2 is notably more responsive to stimuli in cognitive experiments and offers a higher signal-to-noise ratio (Niu et al., 2019). Therefore, HbO2 concentration is utilized to assess cognitive load during shifts in decision-making technological paradigms. The raw HbO2 data collected from participants is represented as Beta values.

## 4 Experimental results

In this experiment, a total of 24 sample groups (72 participants, with 3 people per group) were recruited from universities in Shanghai. Among them, 18 groups (54 participants) were eligible for this study. The 54 participants had an average age of 24.22 (SD = 3.62), including 34 males and 20 females. The sample included 25 undergraduates, 21 master’s students, and 8 doctoral students, all of whom had either interned at or were currently working in engineering companies within the past year.

### 4.1 Behavioral results for decision-making

The behavioral results of decision experiments are shown in Table 6. In the individual decision-making stage, the average decision quality of 18 sample groups was 0.9595 (SD = 0.0137). In the group decision-making stage, the average decision quality of them reached 0.9713 (SD = 0.0099). In addition, the average interdisciplinary communicative capability of them in group decision-making stage was 0.7986 (SD = 0.0715).

**Table 6:** The behavioral results of decision experiment.

The statistical results of behavioral data in decision experiment	Mean	SD	Max	Min
Individual decision-making stage				
Decision quality	0.9595	0.0137	0.9834	0.9331
Group decision-making stage				
Decision quality	0.9713	0.0099	0.9913	0.9583
Interdisciplinary communicative capability	0.7986	0.0715	0.9058	0.6455

The average decision quality increases and the fluctuation of decision quality decreases from individual decision-making to group decision-making, as shown in Table 6. It indicates an improvement in the quality of decision after participants moving from individual decision-making to group decision-making paradigm. Less fluctuation in the statistics of decision quality, suggesting that the distribution of decision quality for group decision-making paradigm is more concentrated.

#### 4.1.1 Change of decision quality under decision-making paradigm shift

**Figure 6:**
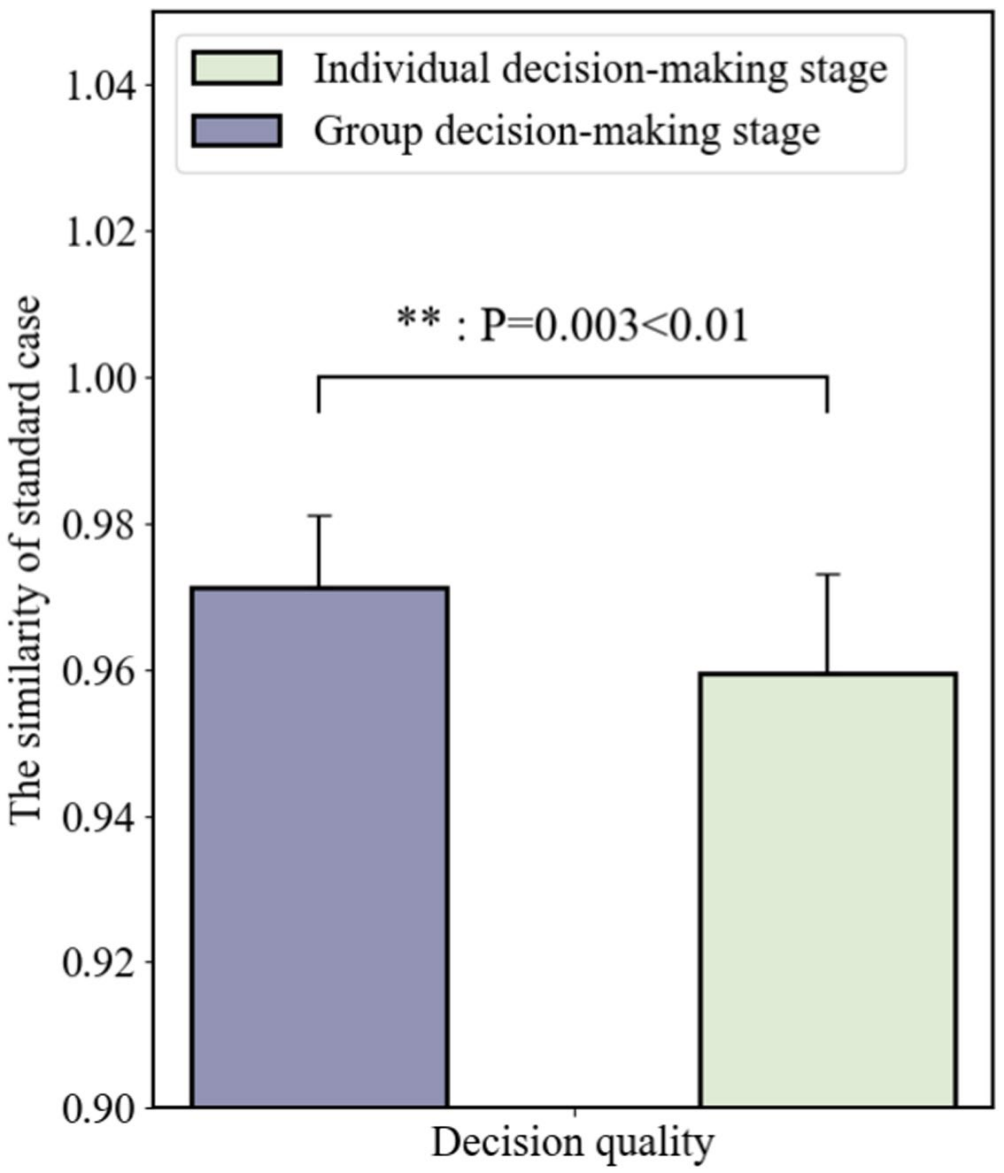
The decision quality under different technological paradigm shifts.

Compared with two decision phares, we find that decision quality $Q_{d}$ of group decision-making is significantly different (t = −3.549, *p* = 0.003 < 0.01), shown in Figure 6. It indicates that the quality of decisions in the group decision-making phase was significantly higher than in the individual decision-making phase, suggesting that group communication significantly improves the quality of decisions (better than single-discipline individual decision-making) under the designed MADM- LGD group decision-making paradigm.

#### 4.1.2 Different prior cognitive level of participants

According to different prior cognitive levels of participants, the participants were relatively divided into two groups—high cognitive level (top 50%, 27 people-9 groups) and low cognitive level (bottom 50%, 27 people-9 groups).

The prior cognitive level orientated questionnaire used in this study contains 10 constructs as shown in Table 7. Cronbach’s alpha coefficients of the scale on all 10 constructs are greater than 0.7, which indicates that the scale has high intrinsic reliability in assessing subjects’ prior cognitive level of decision making.

**Table 7:** The Cronbach’s alpha coefficients of the scale.

Indicator	Cronbach’s Alpha	Item count
Willingness to innovate and explore new solution	0.895	5
Interdisciplinary team leadership	0.777	2
Resilience in multidisciplinary team decision-making	0.869	3
Professional competence and professional awareness	0.825	2
Willingness to share knowledge	0.861	3
Ability to integrate knowledge across multiple disciplines and fields	0.859	2
Trust in other members of the group decision-making team	0.887	3
Team communication and coordination skills	0.900	5
Willingness to work as a team	0.912	5
Team conflict management skills	0.724	2

**Figure 7:**
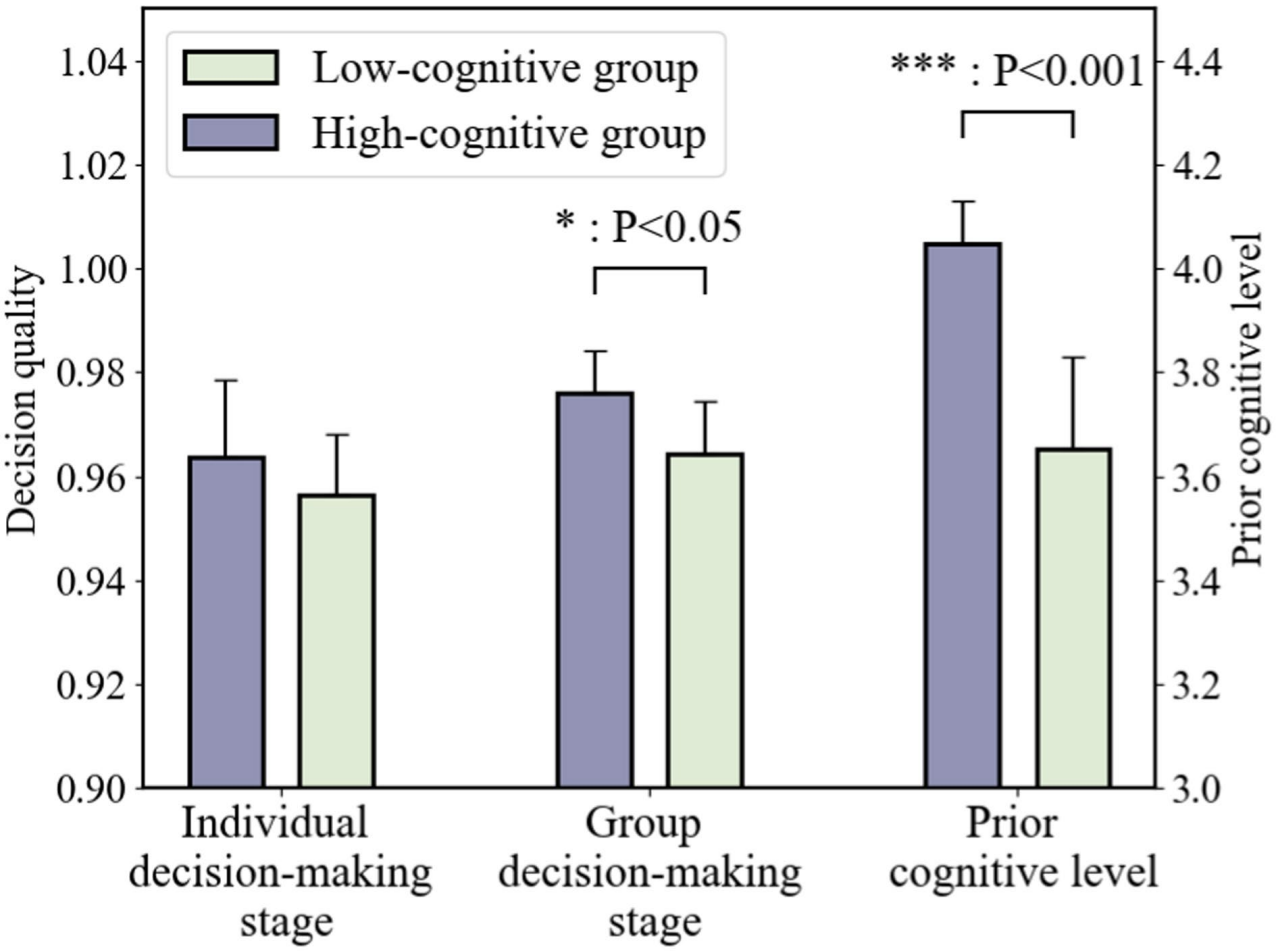
The decision quality under different prior cognitive level of participants.

As shown in Figure 7, compared with two groups for prior cognitive level, we find that $P_{\text{co}}$ is significantly different (t = −10.844, *p* = 0.000005 < 0.001). In the individual decision-making parse, the difference of $Q_{d}$ was not significant (t = −0.987, *p* = 0.353 > 0.05). While in group decision-making stage, $Q_{d}$ of high prior cognitive groups is significantly higher (t = −3.046, *p* = 0.016 < 0.05) than low prior cognitive groups. It indicates the fact that high cognitive groups can effectively communicate and communicate across disciplines, reduce conceptual barriers and heterogeneity across disciplines, and promote common conceptual understanding.

#### 4.1.3 Different interdisciplinary communicative capability of participants

According to different interdisciplinary communicative capabilities of participants, the participants were relatively divided into two groups—high capability level (top 50%, 27 people-9 groups) and low capability level (bottom 50%, 27 people-9 groups).

**Figure 8:**
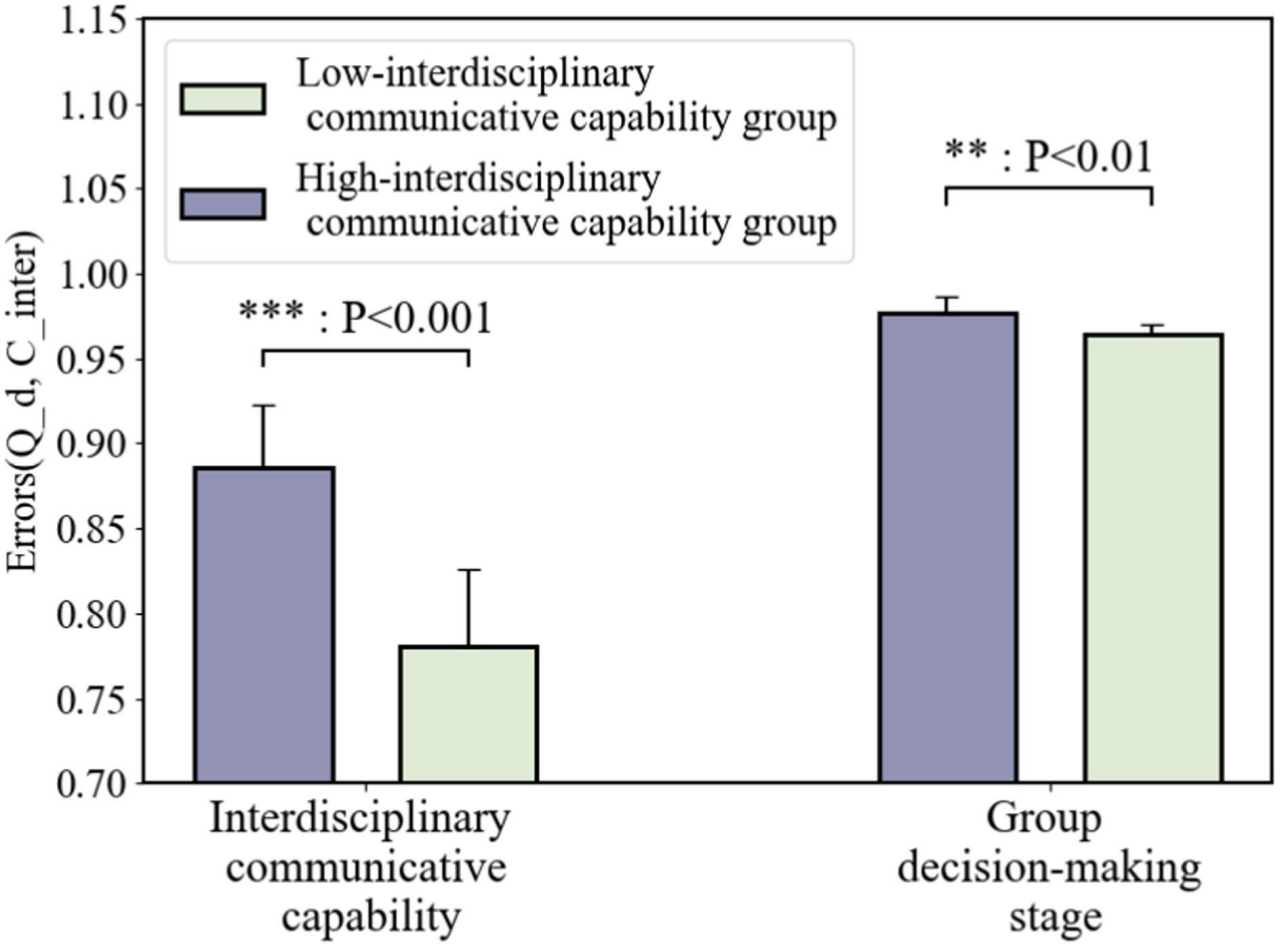
The decision quality under different interdisciplinary communicative capabilities of participants.

As shown in Figure 8, compared with two groups for interdisciplinary communicative capability, we find that $C_{\text{inter}}$ is significantly different (t = −24.619, *p* = 7.92E−09 < 0.001). $Q_{d}$ of high interdisciplinary communicative capability groups is significantly higher (t = −3.575, *p* = 0.007 < 0.01) than low interdisciplinary communicative capability groups. It suggests that the quality of group decision-making is better in groups that communicate effectively across disciplines than in groups that do not communicate effectively across disciplines. This demonstrates, in terms of statistical significance, the need for interdisciplinary group decision-making to facilitate the interpretation and communication of heterogeneous engineering semantics and to promote common understanding.

#### 4.1.4 Joint regression analysis

The results of the joint analysis in Table 8 demonstrate that the joint linear regression of $P_{\text{co}}$ and $C_{\text{inter}}$ on $Q_{d}$ has a high degree of explanation of 0.7163, and from the Partial R-squared in Table 9, there is multicollinearity between $P_{\text{co}}$ and $C_{\text{inter}}$.

**Table 8:** The results of regression analysis.

Independent variable	Dependent variable	R-squared
$P_{\text{co}}$ and $C_{\text{inter}}$	Group $Q_{d}$	0.7163
$P_{\text{co}}$	Group $Q_{d}$	0.7022
$C_{\text{inter}}$	Group $Q_{d}$	0.7038

**Table 9:** Partial R-squared of linear regression.

Variable excluded from multi-regression	Partial R-squared
$P_{\text{co}}$	0.0421
$C_{\text{inter}}$	0.0472

The Pearson correlation coefficient between $P_{\text{co}}$ and $C_{\text{inter}}$ is 0.9630. Based on the above analysis, we can deduce that $P_{\text{co}}$ and $C_{\text{inter}}$ exhibit a strong linear positive correlation. Specifically, the participant’s subjective prior capability ($P_{\text{co}}$) is positively associated with their interdisciplinary communication ability ($C_{\text{inter}}$) displayed during group decision-making. This conclusion reflects the mapping relationship from subjective prior knowledge to objective behavior. It further supports the statistical analysis of prior cognition (shown in Figure 7), indicating that prior cognitive levels do not have a significant impact during the individual decision-making phase but play a significant role during the group discussion phase. The level of interdisciplinary communication serves as a mediating behavioral variable, mapping the prior cognitive level to the quality of group decision-making. The prior cognitive level influences the decision quality by affecting the participant’s objective interdisciplinary communication behavior.

### 4.2 Neurophysiological results under decision-making paradigm shift

#### 4.2.1 Mapping of brain area and analysis of significant activation

To study the cognitive load changing in the different regions of the prefrontal cortex during the decision-making paradigm shift, we collected Oxy-Hb concentration data of all valid participants. Mean Oxy-Hb concentration (*β* value after NITS_KIT treatment) was assigned as the evaluation index of cognitive load during knowledge transfer, as shown in Table 10.

**Table 10:** The statistical results of Oxy-Hb concentration.

	Mean(*10^-5^)	SD (*10^-4^)	Max (*10^-3^)	Min (*10^-3^
meanBeta_rest1	−0.0548	0.0831	0.0404	−0.0543
meanBeta_I1	0.0830	0.2429	0.1394	−0.2120
meanBeta_I2	−0.1486	0.1502	0.0794	−0.0876
meanBeta_rest2	0.1190	0.1265	0.0926	−0.0759
meanBeta_G1	−0.0118	0.3044	0.1740	−0.1611
meanBeta_G2	0.1195	0.1221	0.0784	−0.0570
meanBeta_G3	0.1407	0.1254	0.0632	−0.0602
meanBeta_G4	0.0970	0.0992	0.0636	−0.0463
meanBeta_G5	0.1056	0.2272	0.2543	−0.1507

Heat maps of the brain area with significant activation in different decision-making paradigms are shown in (shown in Appendix A). By One-sample T-test on β value, the detection value is 0, and the *p*-value is corrected by BHFDR. The statistics of significantly activated channels during the individual decision-making parse and the group decision-making parse are made respectively, shown in (shown in Appendix B).

In stage I1, the channels that were activated include C4 (t = −4.445, *p* = 3.18E-05) and C12 (t = −3.392, *p* = 0.0011). Moving on to stage I2, the activated channels are C1 (t = 2.465, *p* = 0.0161), C2 (t = 2.421, *p* = 0.0180), C5 (t = 2.130, *p* = 0.0367), C8 (t = 2.640, *p* = 0.0102), C21 (t = 2.926, *p* = 0.0046), and C22 (t = 2.602, *p* = 0.0113). During stage G1, channels C2 (t = 3.067, *p* = 0.0031), C8 (t = 3.052, *p* = 0.0032), and C21 (t = 3.961, *p* = 0.0002) are activated. In stage G2, the sole activated channel is C21 (t = 4.387, *p* = 3.91E-05). For stage G3, the activated channels are C1 (t = 2.748, *p* = 0.0076), C2 (t = 2.920, *p* = 0.0047), C5 (t = 2.932, *p* = 0.0045), C8 (t = 3.322, *p* = 0.0014), C21 (t = 4.628, *p* = 1.62E-05), and C22 (t = 2.753, *p* = 0.0075). Stage G4 sees the activation of channel C21 (t = 3.578, *p* = 0.0006). Finally, in stage G5, the activated channels are C4 (t = −2.881, *p* = 0.0052), C8 (t = 2.883, p = 0.0052), and C21 (t = 3.599, p = 0.0006). Significantly activated channels are also referred to as regions of interest (ROI). As we can see in (shown in Appendix B), all the significantly activated channels are in 10-FOA (frontopolar area).

#### 4.2.2 Mean oxy-Hb concentration under different stages

**Figure 9:**
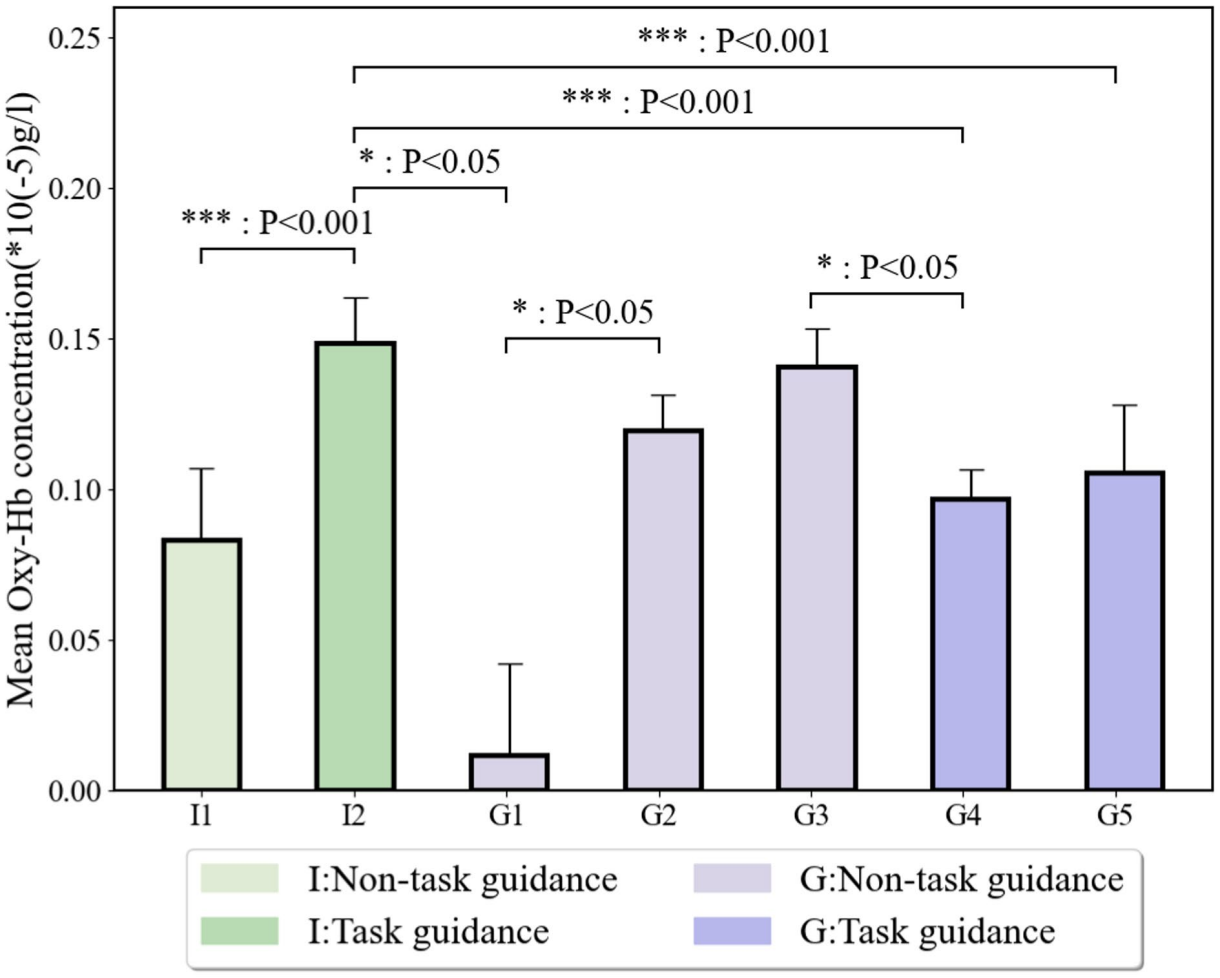
Mean Oxy-Hb concentration under different stages.

As shown in Figure 9, significant differences in mean Oxy-Hb concentration are observed across sequence stages. Specifically, after completing stages I1 and I2, a notable difference was found (t = 3.957, *p* = 0.000079 < 0.001), with the mean Oxy-Hb concentration in I2 being higher than that in I1. Similarly, upon finishing I2 and G1, significant differences were recorded (t = −1.965, *p* = 0.049 < 0.05), with I2 exhibiting higher mean Oxy-Hb concentration than G1. The comparison between G1 and G2 also revealed significant differences (t = −1.993, *p* = 0.046 < 0.05), with G2 showing higher mean Oxy-Hb concentration than G1. After completing G3 and G4, a significant difference was again observed (t = 2.348, *p* = 0.019 < 0.05), with G3 having a higher mean Oxy-Hb concentration than G4.

In the task-guidance stages, significant differences were noted when participants finished I2 and G4 (t = −9.014, *p* = 5.59E−19 < 0.001), with I2 showing a higher mean Oxy-Hb concentration than G4. Likewise, when transitioning from I2 to G5, significant differences were found (t = −4.962, *p* = 0.00000077202 < 0.001), with I2 having a higher mean Oxy-Hb concentration than G5.

#### 4.2.3 Regression analysis of behavioral and neural data

The Pearson analysis results indicated that $P_{\text{co}}$ was linearly unrelated (r = 0.249, *p* = 0.335) to neurocognitive load in the individual decision-making phase, whereas it showed a highly linear negative correlation (r = −0.492, *p* = 0.045) with neurocognitive load in the group decision-making phase.

This is consistent with the results of 4.1.2 for the statistics on prior cognitive level: level of prior cognition plays a role only in the group decision-making phase, reducing cognitive load and improving the quality of group decisions. $C_{\text{inter}}$ shows a highly negative correlation (r = −0.510, *p* = 0.037) with neurocognitive load: the ability to communicate and communicate across disciplines significantly reduces neurocognitive load.

All analyzed above is consistent with the theoretical pathway constructed: “Semantic heterogeneity → cognitive conflict → cognitive load → decision bias.” This explains at the neural level that high interdisciplinary communication capabilities can reduce cognitive load and then improve decision quality.

## 5 Discussion

Combined with the four hypotheses mentioned above, we discussed the mechanism and cognitive factors of the Interdisciplinary Group Decision-Making in the proposed MADM-LGD.

### 5.1 Prior cognitive level impacts the decision performance

Figure 7 shows that the level of prior cognition had a positive effect on the quality of decisions in the interdisciplinary group decision-making phase. Therefore, hypothesis H2b cannot be rejected, i.e., if interdisciplinary teams have different levels of prior cognition, then there will be a significant difference in the quality of interdisciplinary group decision-making results. It is worth mentioning that there was no significant difference in the decision-making results of the individual decision-making stage with silence without communication in the case of teams with different levels of prior cognition, so hypothesis H2a is rejected.

It is noteworthy that we found an underlying law on how the level of prior cognition affected the decision quality: Groups with high levels of prior cognition need to undergo interdisciplinary communication in order to exert the high level of prior cognition to improve the quality of group decision-making, and that high level of prior cognition does not positively affect the final quality of decision-making in the individual decision-making stage of silent non-communication.

### 5.2 Interdisciplinary communicative capability impacts the decision performance

From 5.1, we know that interdisciplinary teams with a high level of prior cognition can significantly improve the quality of decision-making through communication in the group decision-making phase. The $C_{\text{inter}}$ indicator is designed to focus on the “semantic interaction of interdisciplinary teams in the group decision-making phase of engineering,” the definition of which is shown in 3.5.3, and is an indicator that evaluates the adequacy of semantic communication between disciplines and phases of an interdisciplinary team’s decision-making process. Figure 8 shows that interdisciplinary communication capability has a positive impact on the quality of decision-making during the group decision-making phase of an interdisciplinary team. Therefore, hypothesis H3 cannot be rejected, i.e., if interdisciplinary teams have different interdisciplinary communication capabilities, then there are significant differences in the quality of interdisciplinary group decision-making outcomes. This suggests that interdisciplinary group decision-making needs to facilitate the interpretation and communication of heterogeneous engineering semantics to promote common understanding. Those teams that adequately communicate across disciplines and stages of engineering semantics make better decisions and get better decision results.

### 5.3 Differences in decision performance and neurocognitive differences under the decision paradigm shift

Figure 6 shows that decision quality in the group decision-making stage was significantly higher than that in the individual decision-making stage, which indicated that **H1** cannot be rejected, i.e., interdisciplinary teams can significantly improve the quality of decisions through our designed MADM-LGD paradigm.

Further, we investigate the change in neurocognitive load of interdisciplinary teams under the decision-making paradigm shift. Figure 9 shows that the difference in cognitive load between the individual cognitive phase (I1-reading material only and not task-oriented) and the individual decision-making phase (I2- task oriented) was significant, and the cognitive load of the individual decision-making phase containing the task-oriented phase was larger than that of the non-task-oriented individual cognitive phase. The difference in cognitive load between the individual decision-making stage (I2- task orientated) and the group cognition stage (G1- non-task orientated) is significant, and the cognitive load of the individual decision-making stage containing the task orientated stage was greater than that of the non-task orientated group cognition stage. The difference in cognitive load between the group analysis stage (G2- task orientated) and the group cognition stage (G1- non-task orientated) was significant, and the group analysis stage had a greater cognitive load than the group cognition stage. The difference in cognitive load between the group selection stage (G4-task orientated) and the group analysis stage (G3-non-task orientated) was significant, and the task-containing group selection stage had a greater cognitive load than the non-task orientated group cognition stage. Therefore, hypothesis H4a cannot be rejected, i.e., the cognitive load of the task-orientated stage differs significantly from the non-task-orientated stage for both the individual and group decision-making stages, and the task-orientated stage always has a higher cognitive load than the non-task-orientated stage. Figure 9 shows that there was a significant difference between the cognitive load of the individual decision-making stage (I2-task oriented) and the group selection stage (G4-task oriented), and the individual decision-making stage had a higher cognitive load than the group choice stage. The difference in cognitive load between the individual decision-making stage (I2-task oriented) and the group scoring stage (G5-task oriented) was significant, and the cognitive load was greater in the individual decision-making stage than in the group scoring stage. Therefore, hypothesis H4b cannot be rejected, i.e., there is a significant difference between the cognitive load of the task-oriented stage of individual decision-making and the task-oriented stage of group decision-making, and the cognitive load of the task-oriented stage of individual decision-making is always higher than that of the task-oriented stage of group decision-making.

The results can be distilled into a fundamental principle: task-oriented phases impose a higher cognitive load than non-task-oriented phases, and interdisciplinary teams must accommodate an elevated cognitive burden when performing scoring judgments and making choices. Heterogeneous engineering semantics can induce confusion during individual decision-making, thereby increasing cognitive load; in contrast, interdisciplinary group decision-making alleviates this burden by distributing the pressure associated with disciplinary heterogeneity and fostering a shared, multidisciplinary understanding, which in turn streamlines the group decision-making process.

Notably, our analysis uncovered a distinct pattern within the interdisciplinary group decision-making process. When groups initially engage in discussions of nomenclature parameters and sub-problems characterized by high disciplinary heterogeneity, the communication phase substantially elevates the group’s cognitive load (with stage G2 demonstrating a significantly higher load than stage G1). However, as the discussion evolves and the overarching problem is segmented into smaller, more manageable components, the interdisciplinary team progressively clarifies the relative merits and drawbacks of various parameters and option details. This gradual dissolution of disciplinary heterogeneity leads to a reduction in the overall cognitive load, ultimately enabling the group to converge on a unanimous decision, as evidenced by the significantly lower cognitive load observed in stage G4 compared to stage G3.

### 5.4 Neurological explanation

Table 11 shows the responses of different prefrontal regions to cognitive loads. It supports that reducing cognitive load in prefrontal regions such as FOA, PTBA, DLPFC, and IPFG helps to enhance task performance, increase cognitive control, reduce emotional disturbances, and promote learning efficiency and psychological recovery (Spijkerman et al., 2024; Era et al., 2020).

**Table 11:** Responses of different prefrontal regions to cognitive loads.

ROI	Main function	Performance under different cognitive load
FOA	Higher-order abstraction integration, multitasking	As cognitive load rises (e.g., multidimensional integration tasks), the anterior polar cortex is continuously activated to process abstract associations and future goals (Kroger and Kim, 2022).
PTBA	Semantic Selection and Suppression of Interference	Continuous activation of this region for semantic suppression and selection in tasks with high contextual interference (e.g., prosodic interference) (Barredo et al., 2016)
DLPFC	Working memory, cognitive control, attention	Extremely sensitive to task load: the higher the cognitive load, the more active the DLPFC is, especially in tasks such as executive control and working memory updating (Jung et al., 2021).
IPFG	Inhibition control, reaction selection	Its involvement in selection in cognitive control showed task load-related activation with language processing tasks, especially in response conflict tasks (Mitchell et al., 2009).

### 5.5 Suggestions for HI and AI

#### 5.5.1 Suggestions for interdisciplinary decision teams in knowledge-intensive enterprises (HI)

Based on the discussion mentioned above, four suggestions are proposed for interdisciplinary decision groups to improve decision quality and fully utilize human intelligence in knowledge-intensive enterprises.

- *Interdisciplinary decision-making needs more communication*: H1 shows that interdisciplinary teams should be able to fully incorporate the opinions of all disciplines into the decision-making process for a comprehensive assessment of decision-making. It is worth noting that the atmosphere of group discussion and exchange should be fair, inclusive and open, fully ensuring that individuals can express their views and opinions normally.
- *Accumulation of experience in daily decision-making*: H2 shows that individuals participating in interdisciplinary group decision-making should fully accumulate disciplinary knowledge of their own field of specialization and experience in decision-making communication in their daily work and decision-making tasks, to fully ensure that they are able to communicate and discuss efficiently with other individuals when participating in interdisciplinary group decision-making tasks.
- *Focus on communication of heterogeneous engineering semantics*: H3 shows that interdisciplinary decision-making teams should adequately communicate key heterogeneous engineering semantics across disciplines to promote common understanding. Therefore, for individuals involved in interdisciplinary decision-making, they should fully improve their professionalism and ability to express themselves in a generalized way to ensure that participants from other disciplines can understand during the communication process.
- *Multi-stage discussion*: The results of the discussion in H4 guide the process paradigm of interdisciplinary group decision-making. Therefore, the interdisciplinary decision-making team should carry out multiple rounds of discussion by stages when communicating, break down the difficult interdisciplinary problems into easier single-discipline problems, and fully grasp the purpose of the various stages of the discussion to carry out efficient discussion. This can effectively reduce the cognitive load of the group and improve the quality of decision-making.

#### 5.5.2 Suggestions for group decision-making of AI agents (AI)

Based on H4 and H3, the MADM-LGD experimental paradigm we designed can guide the iterative process paradigm for AI multi-agent group decision-making and prompt word design. Table 12 presents a new iterative optimization paradigm for joint decision-making by multidisciplinary domain expert agents. It focuses on enhancing interdisciplinary communication among agents during the group decision-making process, reducing disciplinary barriers related to the target task, fostering integrated understanding, and facilitating decision-making.

**Table 12:** The design of a cognition-inspired AI multi-agent decision-making paradigm.

The design of a cognition-inspired AI multi-agent decision-making paradigm.
*Input*: agent $A_{i}\in A$, $A_{i}$, the discipline category $K_{i}\in K$, corresponding to $A_{i}$. 1: Decompose interdisciplinary grand challenges into disciplinary subproblems in K.
2: For each $K_{i}\in K$:
3: For each $A_{i}\in A$:
4: if $A_{i}=K_{i}$:
5: $A_{i}$ elaborates on the terminology, concepts, and parameters for subproblem $K_{i}$.
6: $\left(A-A_{i}\right)$ repeat the terminology, concepts, and parameters for subproblem $K_{i}$.
7: Agent $A_{i}$ verifies whether the representations of other agents are correct. (If it is incorrect, return step 5. If correct, then iteration.) 8: End if. 9: End for. 10: End for. 11: Compare and discuss the advantages and disadvantages of the parameters.
12: Discuss collectively and decide jointly.

### 5.6 Implications and contributions

The main implications and contributions are summarized in two aspects.

- *A modified experimental paradigm (MADM-LGD) for interdisciplinary group decision-making.* This study improved multi-attribute decision-making tasks (MADM) by proposing a multi-attribute decision-making paradigm based on leaderless group discussion (LDG), referred to as MADM-LGD, and introduced an experimental method for decision paradigm transition. Experimental results show that the designed MADM-LGD paradigm can significantly improve the quality of group decision-making. This paradigm is applicable to both knowledge-intensive enterprise human expert decisions and multi-expert agent decisions in the field of artificial intelligence, providing a general paradigm for interdisciplinary group decision-making.
- *An indicator to measure the interdisciplinary communication capability.* $C_{\text{inter}}$ indicator is designed to assess the “semantic interaction among interdisciplinary teams during the group decision-making phase of engineering.” It serves as a measure of how effectively semantic communication is facilitated across different disciplines and stages in the decision-making process of an interdisciplinary team.

## 6 Conclusion and future work

This study explored how prior cognitive levels and interdisciplinary communication ability influence decision-making quality from the perspectives of neuroscience and cognitive psychology. Additionally, it investigates the changes in group decision-making performance and cognitive load under decision paradigm shifts. The results show that: (1) prior cognitive levels have no significant effect on decision quality during the individual decision-making phase, but they positively influence decision outcomes during the group decision-making phase; (2) interdisciplinary communication ability positively impacts decision performance, with teams possessing higher interdisciplinary communication ability achieving better group decision-making results; (3) the task-oriented phase has a higher cognitive load compared to the non-task-oriented phase, and interdisciplinary group decision-making helps reduce the team’s cognitive load, alleviating the cognitive pressure of heterogeneous engineering semantics, promoting multidisciplinary mutual understanding and further improving decision quality.

Limitations of this study are essential to note: (1) It is undeniable that subjective willingness to share and communicate may lead to a shift from leaderless group discussion to leader-led group discussion and further affect group decision-making performance. Therefore, future work could focus on participants with different subjective communication willingness and examine whether there are significant differences in brain regional activation levels. (2) Issues related to cognitive control and visual attention regulation have not been fully explored. This study only examines brain activity and cognitive load using fNIRS technology. Future research could conduct multimodal experiments combining fNIRS and eye-tracking to further explore the neural basis of interdisciplinary group decision-making.

## Data Availability

The original contributions presented in the study are included in the article/Supplementary material, further inquiries can be directed to the corresponding author.
