# Supplementary material for: Disciplinary barriers need communication: a behavioral and fNIRS study under group decision-making paradigm shift based on cabin design
Source: Front Neurosci. 2025 May 20;19:1594111. doi: 10.3389/fnins.2025.1594111 (PMC12129914; doi:10.3389/fnins.2025.1594111)

# **Appendix A - Heat maps of the brain area with significant activation in different decision-making paradigms**


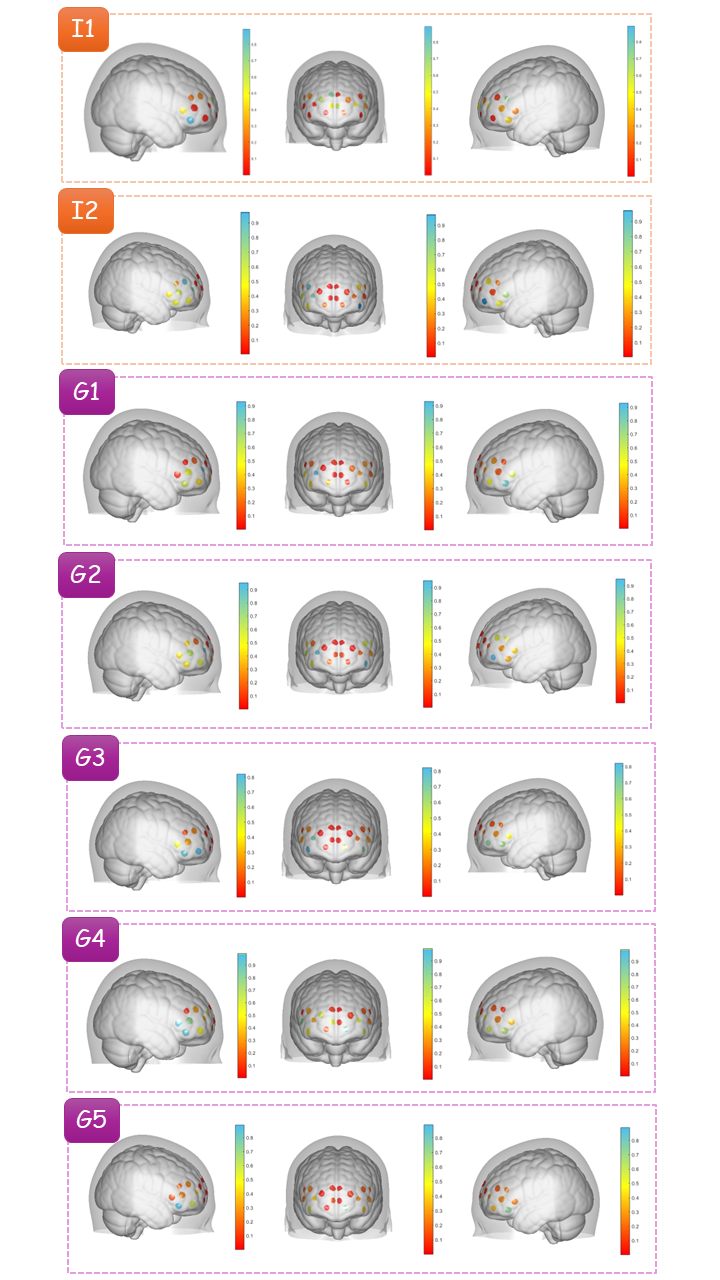

Supplement: Supplementary file 1 [file Table_1.docx]
