# Supplementary material for: Disciplinary barriers need communication: a behavioral and fNIRS study under group decision-making paradigm shift based on cabin design
Source: Front Neurosci. 2025 May 20;19:1594111. doi: 10.3389/fnins.2025.1594111 (PMC12129914; doi:10.3389/fnins.2025.1594111)
Supplement: Supplementary file 2 [file Table_2.docx]

# **Appendix B - The statistics of significantly activated channels**

Table 13 The statistics of significantly activated channels

| Activation channel | T-test statistic value | P-value |
| --- | --- | --- |
| Stage-I1 |  |  |
| C4 | -4.445 | 3.18E-05 |
| C12 | -3.392 | 0.0011 |
| Stage-I2 |  |  |
| C1 | 2.465 | 0.0161 |
| C2 | 2.421 | 0.0180 |
| C5 | 2.130 | 0.0367 |
| C8 | 2.640 | 0.0102 |
| C21 | 2.926 | 0.0046 |
| C22 | 2.602 | 0.0113 |
| Stage-G1 |  |  |
| C2 | 3.067 | 0.0031 |
| C8 | 3.052 | 0.0032 |
| C21 | 3.961 | 0.0002 |
| Stage-G2 |  |  |
| C21 | 4.387 | 3.91E-05 |
| Stage-G3 |  |  |
| C1 | 2.748 | 0.0076 |
| C2 | 2.920 | 0.0047 |
| C5 | 2.932 | 0.0045 |
| C8 | 3.322 | 0.0014 |
| C21 | 4.628 | 1.62E-05 |
| C22 | 2.753 | 0.0075 |
| Stage-G4 |  |  |
| C21 | 3.578 | 0.0006 |
| Stage-G5 |  |  |
| C4 | -2.881 | 0.0052 |
| C8 | 2.883 | 0.0052 |
| C21 | 3.599 | 0.0006 |
